# Supplementary material for: Effect of guided dual-sensory information on motor learning outcomes based on spatiotemporal dimensions
Source: PLoS One. 2025 Nov 20;20(11):e0337236. doi: 10.1371/journal.pone.0337236 (PMC12633867; doi:10.1371/journal.pone.0337236)
Supplement: Attachment — Submitted filename: reviewing_plos_one.docx [file pone.0337236.s001.docx]

Overall, this is a promising study with valuable contributions to understanding the role of instructional information in motor learning. However, it is recommended to revisit the methodology and the presentation of the results, as they are not easy to follow in their current form. A clearer and more structured explanation would help improve the readability and comprehensibility of the findings, making it easier for readers to follow the study's logic and conclusions.

**Abstract**

Line 2 : « for enhancing”… or “to enhance”

Line 5 : The concept of 'Movement Staging' is not very clear. It would be helpful to provide a more detailed explanation or clarification on what exactly this term refers to and how it is applied in this context.

Line 7: "3" should be written as "three" in formal writing.

Line 9: "3" should be written as "three" in formal writing.

Line 11: Use "In terms of timing” to replace “temporally”

Line 13 "Spatially, upper-limb motor learning required more guidance than lower-limb motor learning."

Inferential statistics must be reported in the results section.

**Introduction**

The introduction presents the context well in terms of theoretical and empirical approaches to motor learning. a few clarifications and modifications are required

Line 26 : Can you illustrate the spatiotemporal dimension of motion?

Line 28 : The research question should not appear here, but rather at the end of the presentation of your theoretical elements

Line 35: present the role of information in motor learning. What are they used for and when? See perhaps the work on internal models ...

Line 36 : Can you provide an example of the importance of external (and internal) information in the execution of movements and in learning, based on the literature?

**Method**

The method needs to be rewritten as it is currently unclear. It should be reorganized following this outline:

- **Population :** Add gender distribution
- **Experimental Design**: Specify the three conditions... Line 163-164: it is not clear.
- **Materials**: Describe what is common between the three videos as well as what distinguishes them. What is shown in each video? What instructions are provided? What are the markers? What is explained from line 172 to 179 should appear in this paragraph.
- **Procedure**: “Participants were randomly assigned to one of the three groups. Each participant watched a video corresponding to their group and then completed a motor cognition and performance test…” Include details such as the number of sessions, duration, etc . Explain which evaluation scales were taken and at what time.

A diagram with evaluation and intervention to illustrate the procedure would be helpful.

Line 163 : the same sentence twice.

Line 215: Feedback – What information is provided?

**Learning outcome data collection**

Line 221 : add after motor cognition “(Set Shot Motor Cognition Test)”

Line 222: Set Shot Motor Cognition Test – Did you create this scale? If so, could you provide examples of the items? Who completes it?

Line235: add after movement pattern performance “(Set Shot Movement Pattern Rating Scale”)

Line 236: Set Shot Movement Pattern Rating Scale – Where does this scale come from?

Line 245: Interrater reliability – Which reference are you relying on to justify that the reliability is good?

**Data analyses**

If I understand correctly, you performed an ANOVA at baseline and then another ANOVA at post-intervention. Why did you not conduct a one-way ANOVA with repeated measures and a factor of group: TIME (pretest vs posttest vs retention) x GROUP (video vs video with narration vs video with narration and markers)? It seems important, when discussing learning, to include a comparison between the data before and after the intervention (in addition to the comparison between groups).

**Results** :

Please report the effect sizes.
It would seem more appropriate to report all the effects in the text and illustrate them with a graph rather than using tables.

Line 288 replace “=0.00” by “p<0.001”

**Tables**

The tables are not presented in the same way, and the groups are not always named consistently . Please standardize them (If you choose to keep the tables).
The post-hoc statistics are not reported (they should be included in the text).

**Discussion**

The discussion is generally clear but it would benefit add more connections between results of this study and some studies suggest contradictory findings:

- It would be beneficial to explicitly address why these discrepancies exist—such as differences in methodology, participant characteristics (age), or the type of skills being learned.
- The interpretation of results could be clarified. For instance, when mentioning that adding markers "did not create a redundancy effect," can you explain this reported redundancy effects with multimedia learning.
- The statement that "markers highlight key details, which assist learners in establishing memory schemas and improves error detection" is interesting, but it would benefit from additional supporting evidence or references to studies that have shown these specific cognitive processes in motor learning.
- It might be relevant to discuss the fact that in this study these are novices. The results may be different for experts ....
