# Supplementary material for: Effect of guided dual-sensory information on motor learning outcomes based on spatiotemporal dimensions
Source: PLoS One. 2025 Nov 20;20(11):e0337236. doi: 10.1371/journal.pone.0337236 (PMC12633867; doi:10.1371/journal.pone.0337236)
Supplement: Attachment — Submitted filename: PLOS Reviewer Comments__PONE-D-25-06505_AP.docx [file pone.0337236.s002.docx]

This manuscript investigates how different multimedia instructional methods (specifically visual only, visual plus auditory, and guided dual sensory using visual and auditory information with markers) affect motor learning of the basketball set shot among middle school students. The authors use a spatiotemporal analysis framework to evaluate both movement pattern performance and motor cognition across different stages of movement and various limb segments. The study is timely and addresses an important question in physical education and motor learning: how can digital instructional tools be optimized for teaching complex motor skills?

The study is methodologically sound, with a clear experimental design, a substantial sample size (N = 132), and careful measurement of both cognitive and physical aspects of motor learning. The use of retention testing and inter/intra-rater reliability measures further strengthens the rigor of the work. The application of a spatiotemporal lens to instructional effectiveness is a novel and meaningful contribution to the field.

That said, I believe the manuscript requires **major revisions** prior to consideration for publication. The manuscript would benefit from clearer writing, greater theoretical integration with the presented motor learning frameworks, and a deeper discussion of findings, limitations and generalizability.

**Major Concerns:**

**1. Lack of a cohesive theoretical framework in the Introduction**

While the authors reference several relevant learning theories (e.g., dual coding theory, cognitive load theory, multimedia learning), these frameworks are introduced in a fragmented way. The Introduction feels like a collection of loosely connected concepts rather than a synthesized argument leading to the present study. As a result, it is unclear how the theoretical background specifically motivates the study design or outcome measures.

In addition, a clear rationale for why the basketball set shot was chosen as the target skill is missing. Although it is noted as a common scoring technique, the authors do not explain why this particular skill is ideally suited for examining dual-sensory instructional methods or spatiotemporal learning. For example, is the set shot particularly reliant on visual modeling? Does it break down neatly into movement stages and limb segments more than other skills would? Establishing this would strengthen the case for generalizability and relevance.

Lastly, a central hypothesis or set of testable predictions is not explicitly stated. This makes it difficult to discern what the authors expected and how the results support or challenge their assumptions.

**2. Insufficient methodological details**

The study does not provide adequate information about participant demographics beyond age (e.g., gender distribution, physical literacy or motor skill baseline beyond set shot experience). This is especially important given that sex or experience level could potentially influence motor learning or responsiveness to different instructional modalities.

Additionally, the randomization process is not described in sufficient detail. The authors state that participants were randomly assigned to one of three instructional groups, but there is no mention of how this was done (e.g., computer-generated random numbers, stratified sampling, class-based assignment). This limits confidence in the equivalence of groups at baseline.

It is also unclear whether the expert raters were blinded to group allocation during performance assessment. Without blinding, there is risk of observer bias, particularly since scoring was based on qualitative movement ratings.

**3. Ambiguity in describing the guided “marker” cues**

The “guided dual sensory” condition (i.e. the addition of visual markers) is insufficiently described. The manuscript does not clarify what the markers looked like (e.g., flashing frames, arrows, highlights?), where on the screen they appeared (e.g., limb segments, ball), how long they were displayed, or whether they were dynamic (moving) or static. These features are critical to understanding how attention might have been guided.

Moreover, there is no discussion of whether the markers might have introduced unintended visual biases, distracted from important movement features, or created a redundancy effect. Given that attentional guidance is central to the study’s rationale, this lack of detail undermines interpretability and reproducibility.

**4. Statistical concerns**

The authors do not report effect sizes, limiting interpretation of the practical significance of the findings. Additionally, the use of separate one-way ANOVAs may not be appropriate given the within-subject dependency across time. A mixed-effects ANOVA would better account for within-subject variation across timepoints and reduce the risk of Type I error from multiple comparisons. For example: group (between) × time (within).

**5. Lack of discussion of null results**

Several analyses yielded non-significant findings (e.g., between-group differences in posttest accuracy, lower-limb performance), but these are not addressed in the discussion. Ignoring these results limits the reader’s understanding of the full pattern of findings and weakens the credibility of the interpretations. A balanced discussion should acknowledge where the instructional interventions did not yield clear benefits.

**6. Discrepancy between form improvement and performance outcome**

Although the study shows improvements in movement patterns, these did not consistently translate into better shooting accuracy. The discussion does not address this discrepancy. A possible explanation may lie in the early stage of motor learning, where improved form does not yet yield measurable gains in performance outcomes. However, this should be explicitly discussed to clarify the practical implications of the findings.

**7. Interpretations about attention without direct measurement**

The discussion relies heavily on attentional guidance theory to explain the observed benefits of marker-based instruction. However, attention was not directly measured in the study (e.g., through gaze tracking or reported focus), making these interpretations speculative. This limitation should be acknowledged more explicitly, especially since attention is central to the study’s proposed mechanism.

**Minor Revisions:**

Minor revisions are recommended to improve grammar, sentence structure, and overall flow, as several sections contain typographical errors, awkward phrasing, and inconsistent formatting that could hinder readability. See some examples below:

1. Line 34: Verb tense should say ‘enables’

2. Line 164: missing space after the period

3. Line 212: Sentence is the past tense.

4. Line 455 – sentence ends with an unnecessary ‘is’

5. Line 511 – likely a typo ‘studing’ – ‘studying’
