# Supplementary material for: Effect of guided dual-sensory information on motor learning outcomes based on spatiotemporal dimensions
Source: PLoS One. 2025 Nov 20;20(11):e0337236. doi: 10.1371/journal.pone.0337236 (PMC12633867; doi:10.1371/journal.pone.0337236)
Supplement: Attachment — Submitted filename: author revised-O-2024-007700 R1 review1+2.docx [file pone.0337236.s005.docx]

*Dear Reviewer,*

*Thank you for your thorough review of and valuable intellectual input regarding our manuscript. We have carefully addressed each suggestion and revised the manuscript accordingly. We believe these changes respond to your concerns and highlight the educational implications of our study. We sincerely appreciate your insightful recommendations, which contribute to fostering a virtuous cycle of “research informing practice” in physical education. We are more than happy to incorporate any further suggestions to refine our manuscript.*

| *• Black text* | *represents the original comments from the reviewers* |
| --- | --- |
| *• Purple text* | *indicates our direct responses to the reviewers' comments* |
| *• Blue text* | *shows the revised content that corresponds to changes made in the revised manuscript* |

**Reviewer 1**

**1. Lack of a cohesive theoretical framework in the Introduction**

1.1 While the authors reference several relevant learning theories (e.g., dual coding theory, cognitive load theory, multimedia learning), these frameworks are introduced in a fragmented way. The Introduction feels like a collection of loosely connected concepts rather than a synthesized argument leading to the present study. As a result, it is unclear how the theoretical background specifically motivates the study design or outcome measures.

We sincerely appreciate your insightful comments regarding the insufficient integration of theoretical frameworks in our manuscript. Your observation about "fragmented theoretical introduction leading to logical discontinuity" was particularly enlightening. We have systematically restructured the theoretical framework using the Cognitive Theory of Multimedia Learning (CTML) as the unifying thread (specific revisions detailed below). Should any deficiencies remain, we warmly welcome further guidance.

I. Explicitly established CTML as the theoretical foundation in the Introduction, with its dual-channel processing and signaling principles directly supporting the proposed "guiding dual-sensory information" research strategy.

The present study used Mayer’s cognitive theory of multimedia learning (CTML) as its framework. This theory posits that when learners receive information through properly designed visual and auditory channels, this information reduces extraneous cognitive load, promotes deep processing, and enhances motor learning outcomes [5]. On the basis of the dual-channel signaling principle of the CTML [6], this study proposed a visual + audio + markers guided information presentation strategy.（Lines：37-42）

II. The Introduction now presents a theoretically grounded derivation process through Cognitive Load Theory (CLT) and Dual Coding Theory, forming a three-tiered "CTML-CLT-Dual Coding Theory" progressive logic.

Cognitive load theory focuses on how an individual allocates mental resources and suggests that insufficiently accounting for memory limits in instructional design can hinder learning [15,16]. Mayer combined cognitive load theory with dual code theory, creating the Cognitive Theory of Multimedia Learning [17]. Dual code theory suggests that individuals process information through both images and language; that is, learners receive instructional information through different sensory channels [18]. Multimedia instructional design should reduce unnecessary cognitive load and promote understanding of material [5].（Lines：69-74）

III. Incorporating the specificity of motor learning, we employed CTML to clarify the information requirements for action execution, thereby optimizing the delivery methods of multimedia learning information, the scientific validity of content, and the operational appropriateness of movement staging.

Motor learning requires simultaneously processing multichannel information and executing physical movements; these processes compete for limited resources in the learner’s working memory [15, 32].（Lines：100 -104）

The aforementioned findings highlight the need for carefully designed and organized motor learning materials to ensure effective information processing and optimal performance.（Lines：106-108）

Reducing cognitive load during motor learning requires providing information to guide learner attention and facilitate the construction of mental models [15].（Lines：121-122）

IV. When addressing current research limitations, we introduced attention-guiding design requirements based on CLT and CTML's dual-channel/signaling principles. The motor schema theory was proposed to establish foundations for analyzing shooting learning outcomes through spatiotemporal dimensions. Motor learning theories provided the analytical framework for precise assessment of instructional information's impact on participants' motor performance and cognitive representation via quantitative analysis of spatiotemporal data.

Cognitive load theory and the dual-channel with signaling principle of the CTML suggest that motor learning requires matching sensory channel capacity with attention-guiding design [6, 15].（Lines：133-135）

Additionally, Schmidt’s schema theory suggests that phased practice establishes refined motor schemata and enhances movement adaptability [40].（Lines：144-146）

Supplementary references：

40. Schmidt RA. A schema theory of discrete motor skill learning. Psychol Rev. 1975;82(4): 225–260. doi: 10.1037/h0076770

The set shot is a highly visual motor skill [44] that requires learners to establish internal representations by observing the movements, a process consistent with the fundamental assumptions of observational learning and imitation in multimedia learning theory.（Lines：167-170）

V. Both research question derivation and Discussion sections strictly align with theoretical presuppositions. The results respectively validate relevant theories, echoing the problem formulation and inferences in the Introduction to ensure theoretical consistency from hypotheses to conclusions.

This study hypothesized that incorporating verbal narration and visual markers (red flashing frames) in instructional videos would enhance learners’ focus on critical motor learning information, increasing movement pattern performance and motor cognition outcomes. This design is consistent with the core proposition of CLT, which posits that learners most effectively manage limited cognitive resources by focusing on the most task-relevant information.（Lines：189-194）

Guided dual-sensory information aligns with both the dual-channel principle and the signaling principle of CLT [11]. （Lines：606-607）

The findings of this study validate the guided dual-sensory information strategy based on the dual-channel processing and signaling principles of the CTML. （Lines：767-768）

1.2 In addition, a clear rationale for why the basketball set shot was chosen as the target skill is missing. Although it is noted as a common scoring technique, the authors do not explain why this particular skill is ideally suited for examining dual-sensory instructional methods or spatiotemporal learning. For example, is the set shot particularly reliant on visual modeling? Does it break down neatly into movement stages and limb segments more than other skills would? Establishing this would strengthen the case for generalizability and relevance.

Your question demonstrates remarkable expertise, and this submission process has been immensely beneficial, helping me better understand how to present empirical research logic. The selection of set shot as the target skill was primarily based on the following considerations:

I. **Instructional Practice Relevance**: Basketball is a compulsory component of the seventh-grade physical education curriculum in Chinese middle schools; it is widely popular among adolescent populations; and it facilitates the implementation of teaching experiments within regular physical education classes.
II. **Motor Learning Characteristics**: The focus is on gross limb segment fundamentals, offering advantages over fine motor skills (e.g., table tennis); technical evaluation is less constrained by observational limitations; and the movement staging is clearly defined.
III. **Theoretical Compatibility**: It aligns with the observational learning hypothesis of multimedia learning theory, as learners rely on visual demonstrations to establish operational representations; it possesses a distinct spatiotemporal movement structure; and it can be readily decomposed into discrete learning units.

Following your suggestion, we have added relevant descriptions in the main text to clarify: the logical connection between skill selection and the theoretical framework (CTML application scenarios); the scientific basis for movement staging; and the ecological validity of the instructional experiment design. Specific revisions are detailed below.

The set shot is a highly visual motor skill [44] that requires learners to establish internal representations by observing the movements, a process consistent with the fundamental assumptions of observational learning and imitation in multimedia learning theory. Additionally, this skill exhibits a well-defined spatiotemporal movement structure that can be clearly divided into preparation stage and execution stages and principally involves control of the upper-limb, the lower-limb, and the ball-handling. These characteristics render the set shot particularly well suited to examining the effects of movement staging and limb segment information guidance in phased motor skill instruction [44,45]. Finally, the set shot is a fundamental skill in secondary school physical education. Students typically lack experience with this skill, and its stable movement characteristics facilitate standardized assessment and instructional application.（Lines：167-177）

1.3 Lastly, a central hypothesis or set of testable predictions is not explicitly stated. This makes it difficult to discern what the authors expected and how the results support or challenge their assumptions.

Thank you for pointing out the lack of clear hypotheses or testable predictions, which could indeed confuse readers and make it difficult to discern the paper's main focus at the outset. Following this suggestion, we have incorporated the following revisions in the Introduction section: (1) posing specific research questions to stimulate thinking on "which information presentation methods are truly effective for motor learning," and (2) stating testable predictions: "This study hypothesizes that...". Finally, the Conclusion section now explicitly states whether the results support the hypotheses. These suggestions have also enlightened us that adopting a reader-oriented perspective when writing academic papers can significantly improve the clarity of the research problems being addressed.

The recognition of these deficiencies in set shot pedagogy prompted the following research question: Which of the three information presentation methods—visual-only (video), dual-sensory audiovisual (video with narration), and guided dual-sensory instruction (video with narration and markers)—is most effective in motor learning?（Lines：181-184）

This study hypothesized that incorporating verbal narration and visual markers (red flashing frames) in instructional videos would enhance learners’ focus on critical motor learning information, increasing movement pattern performance and motor cognition outcomes. This design is consistent with the core proposition of CLT, which posits that learners most effectively manage limited cognitive resources by focusing on the most task-relevant information.（Lines：189-194）

The findings of this study validate the guided dual-sensory information strategy based on the dual-channel processing and signaling principles of the CTML.（Lines：767-768）

**2. Insufficient methodological details**

2.1 The study does not provide adequate information about participant demographics beyond age (e.g., gender distribution, physical literacy or motor skill baseline beyond set shot experience). This is especially important given that sex or experience level could potentially influence motor learning or responsiveness to different instructional modalities.

Thank you for your suggestions on the details of this study. The basic information of the participants is very important, and we will carefully supplement the relevant content. The gender distribution has been supplemented, and the number of male and female students in the class is basically the same. In this experiment, under the premise of controlling the consistency of teaching process and content, the gender indicators of the subjects were not included in the measurement variables. 2. It should be acknowledged that physical literacy was not measured before the start of this experiment. This teaching class is divided into natural classes. Prior to the start of the experiment, we communicated with the physical education teacher and the homeroom teacher. Based on previous records, there was no significant difference in physical fitness among the three classes. According to the analysis of physical education grades, there is no difference in basic motor skills other than shooting among the three classes. These supplementary contents have been reflected in the research object section of the revised manuscript. We sincerely thank you for pointing out this key methodological issue, which significantly enhances the rigor of the research.

The participants were randomly assigned to one of three instructional conditions: a video only group (n = 44), a video with narration (audiovisual) group (n = 44), and a video with narration and markers (guided dual-sensory instruction) group (n = 44), with Classes A (video) and B (video with narration) each contributing 22 male and 22 female students, and Class C (video with narration and markers) contributing 21 male and 23 female students.（Lines：212-217）

Before the experiment, all participants and their parents/guardians were fully informed of the study procedures and safety considerations, and written informed consent was obtained from both the participants and their parents/guardians. Three experts independently conducted baseline assessments of the participants’ motor skills. As detailed in the Results section, no significant between-group differences were observed in the pretest set shot performance among the three groups (p > 0.05), ensuring sample homogeneity.（Lines：224-229）

During the experimental period, the participants did not engage in any additional basketball activities or any form of basketball skills training outside the study protocol.（Lines：234-236）

2.2 Additionally, the randomization process is not described in sufficient detail. The authors state that participants were randomly assigned to one of three instructional groups, but there is no mention of how this was done (e.g., computer-generated random numbers, stratified sampling, class-based assignment). This limits confidence in the equivalence of groups at baseline.

We fully agree with the process description of supplementing random grouping. Thank you for your patient guidance. You have filled in the gaps in our research from the perspectives of experts and readers, and helped improve this article.

The study school admits students using a proximity-based enrollment policy and maintains regular mixed-gender classes. Under these conditions, the motor abilities of the students in the same grade approximate a normal distribution, satisfying random sampling criteria. We employed cluster sampling, randomly selecting three out of the nine seventh-grade classes. （Lines：208-212）

During the experimental period, the participants did not engage in any additional basketball activities or any form of basketball skills training outside the study protocol.（Lines：234-236）

2.3 It is also unclear whether the expert raters were blinded to group allocation during performance assessment. Without blinding, there is risk of observer bias, particularly since scoring was based on qualitative movement ratings.

You have conducted a very detailed review of this article, and expert raters have blindly tested the grouping information during the evaluation process. I have already supplemented that this part of the content is included in the learning effectiveness data collection section.

The final scores were determined by averaging the ratings of the three experts. The expert raters were blinded to the group assignment information during the evaluation process.（Lines：389-391）

**3. Ambiguity in describing the guided “marker” cues**

3.1 The “guided dual sensory” condition (i.e. the addition of visual markers) is insufficiently described. The manuscript does not clarify what the markers looked like (e.g., flashing frames, arrows, highlights?), where on the screen they appeared (e.g., limb segments, ball), how long they were displayed, or whether they were dynamic (moving) or static. These features are critical to understanding how attention might have been guided.

This valuable feedback has significantly enhanced our presentation of relevant content. We acknowledge that the original description of markers lacked sufficient detail, and based on your suggestion, we have refined the cue presentation method. Relevant research indicates that physical cues are more effective than social cues for procedural knowledge acquisition. Given the unique requirements of motor learning - where the markers themselves should not attract excessive attention - we specifically selected physical cue markers (red flashing frames) and strategically positioned them on-screen to highlight key limb segments requiring observation during shooting execution, as detailed in the revised manuscript.

Additionally, research has indicated that physical marker cues (e.g., flashing arrows) in key information areas effectively guide attention [48]. On the basis of these findings, the guided dual-sensory video materials in this study incorporated localized markers targeting movement essentials, which appeared on corresponding limb segment displays without obscuring critical movement feature areas. The marking method employed low-saturation red flashing frames that appeared synchronously with movement essentials presented through both video and narration. Each marker was presented for 1 second and flashed only once before transitioning to a static red frame. Preliminary experimental results on changes in learning outcomes suggested that the presentation of the markers did not induce unintended visual bias, distract attention from critical movement features, or give rise to redundancy effects.（Lines：277-287）

Supplementary references：

48. Boucheix JM, Lowe RK. An eye tracking comparison of external pointing cues and internal continuous cues in learning with complex animations. Learn instr. 2010;20(2):123-135. doi:10.1016/j.learninstruc.2009.02.015

3.2 Moreover, there is no discussion of whether the markers might have introduced unintended visual biases, distracted from important movement features, or created a redundancy effect. Given that attentional guidance is central to the study’s rationale, this lack of detail undermines interpretability and reproducibility.

Thank you for your important feedback. The markers used in this study have been discussed by experts and instructors, and their appearance, color, and shape design can effectively avoid visual interference, and the marker positions all avoid key action feature areas. The results of the pre experiment verified that learners were able to obtain shooting information in the teaching experiment, and the labeling was basically controlled to guide attention without generating redundant information. It has been explained in the research tool.

On the basis of these findings, the guided dual-sensory video materials in this study incorporated localized markers targeting movement essentials, which appeared on corresponding limb segment displays without obscuring critical movement feature areas.（Lines：278-281）

Preliminary experimental results on changes in learning outcomes suggested that the presentation of the markers did not induce unintended visual bias, distract attention from critical movement features, or give rise to redundancy effects.（Lines：284-287）

**4. Statistical concerns**

The authors do not report effect sizes, limiting interpretation of the practical significance of the findings. Additionally, the use of separate one-way ANOVAs may not be appropriate given the within-subject dependency across time. A mixed-effects ANOVA would better account for within-subject variation across timepoints and reduce the risk of Type I error from multiple comparisons. For example: group (between) × time (within).

The statistical considerations you have proposed not only help us improve the rigor of our research, but also deeply inspire us: when exploring the attention mechanism in physical education teaching, every detail of scientific design - from examining individual differences to choosing statistical methods - may become a key to revealing the cognitive laws of human movement.

We agree that individual variability is an important consideration, but in this study, a modified mixed design analysis of variance (ANOVA) was used because it can simultaneously analyze the interactions between between subject factors (experimental group) and within subject factors (time/repeated measures). Ensure the applicability of Mixed design ANOVA through the following design. Through pre-test verification, there was no significant difference between the groups at baseline, meeting the requirements for inter group balance in mixed design ANOVA. Although mixed effects models can handle individual random variations, the pre-test results of this study confirm that the baselines of each group are consistent (without initial individual ability differences), and mainly focus on fixed effects of inter group and temporal interactions, rather than random slopes between individuals. And report the effect size, interpreting the substantive significance of the results. Therefore, Mixed design ANOVA can effectively answer research questions while controlling for type 1 errors (such as using Bonferroni correction), and is more concise and in line with experimental design logic.

Thank you for your suggestion. Based on your suggestion, we have revised our research method and re analyzed the differences in the performance of fixed-point shooting action types and fixed-point shooting results. We have also written the results section.

A mixed-design ANOVA was employed to examine differences in set shot movement pattern performance and set shot outcome performance across the three information presentation groups (video, video with narration, and video with narration and markers) between the pretest and posttest. When significant group × time interactions were observed, simple main effects analyses were conducted, followed by post hoc comparisons using the least significant difference (LSD) method (α = .05). When nonsignificant interactions were identified, the main effects were analyzed separately using LSD post hoc tests (α = .05). （Lines：408-414）

Most of the content in the Results section（Lines：417-589）

**5. Lack of discussion of null results**

Several analyses yielded non-significant findings (e.g., between-group differences in posttest accuracy, lower-limb performance), but these are not addressed in the discussion. Ignoring these results limits the reader’s understanding of the full pattern of findings and weakens the credibility of the interpretations. A balanced discussion should acknowledge where the instructional interventions did not yield clear benefits.

This is an extremely important reminder regarding the non-significant findings that indeed exist in our analysis. For lower limb movement pattern performance, no significant changes were observed in either the time-by-group interaction or the main effects of time and group factors. Similarly, the retention test results showed no differences among the three groups in movement outcome performance. Your keen observation about these non-significant results has helped us profoundly recognize the necessity of completely reporting all research findings - whether significant or not - and we have supplemented this discussion in the revised manuscript.

When analyzing movement limb segments, this study observed both positive gains and nonsignificant changes—particularly in the lower-limb movement patterns during the preparation and execution stages of shooting. This result may be attributable to (1) the low technical requirements to perform lower-limb actions (e.g., knee flexion or push-off); (2) seventh-graders’ fundamental squatting movements having reached near-automatic, near adult-level neuromuscular control through motor experience, [57], consistent with the principle that foundational skills mature earliest in child motor development. Consequently, the instructional interventions resulted in limited benefits to these well-established basic movements. Hence, no significant differences were observed in the lower-limb learning outcomes across the visual-only (video), audiovisual (video with narration), or guided dual-sensory (video with narration and markers) conditions. This finding also explains the inconsistencies observed in studies regarding the efficacy of multimedia in motor skill learning ; these studies have neither employed standardized information presentation methods nor conducted time- or segment-specific analyses, limiting the accuracy and specificity of their results.（Lines：670-684）

Supplementary references：

57. Payne VG, Larry D. Isaacs. Human motor development: A lifespan approach. Routledge. 2024.

Although dual-sensory guidance temporarily enhanced movement pattern performance, its learning effects may be unstable and exhibit noticeable performance decay during retention testing. This finding suggests that the intervention may have facilitated only the shallow encoding of motor skills. This finding aligns with those of a study [54] indicating that neither multimedia presentation method nor measurement phase substantially enhanced shooting accuracy retention.（Lines：701-706）

**6. Discrepancy between form improvement and performance outcome**

Although the study shows improvements in movement patterns, these did not consistently translate into better shooting accuracy. The discussion does not address this discrepancy. A possible explanation may lie in the early stage of motor learning, where improved form does not yet yield measurable gains in performance outcomes. However, this should be explicitly discussed to clarify the practical implications of the findings.

As you astutely pointed out, the inconsistency between movement pattern improvement and movement result performance precisely reveals the complex transformation mechanism from "quantitative change" to "qualitative change" during motor skill acquisition. In response to this invaluable suggestion, we have restructured the discussion logic: first explicitly acknowledging the asynchrony between form and function in early-stage motor learning, then explaining how higher-order motor performance is constrained by more complex factors.

Motor learning is the process through which individuals achieve persistent changes in motor skills through accumulated practice and experience [4]. Although the guided dual-sensory learning group exhibited considerably superior movement pattern performance than the visual-only group, this advantage did not consistently translate into greater shooting accuracy. This result indicates that although information presentation substantially influenced movement pattern learning, shooting accuracy—a higher-order motor skill —likely requires multisystem integration and thus demands more prolonged, specialized practice to achieve substantial improvements in precision.（Lines：707-714）

The participants were engaged in the earliest stages of shooting learning, in which movement execution is slow and unsteady and improved movement patterns do not translate to increased shooting accuracy, reflecting a form–function dissociation in complex motor skills that is consistent with the predictions of the hierarchical skill acquisition model [39].（Lines：721-725）

**7. Interpretations about attention without direct measurement**

The discussion relies heavily on attentional guidance theory to explain the observed benefits of marker-based instruction. However, attention was not directly measured in the study (e.g., through gaze tracking or reported focus), making these interpretations speculative. This limitation should be acknowledged more explicitly, especially since attention is central to the study’s proposed mechanism.

Your observation regarding this issue is absolutely crucial. Indeed, our study's extensive reference to attention-guiding theory to explain marker effects—without employing direct measurement methods such as eye-tracking or subjective attention scales—does carry inherent speculative risks in theoretical interpretation. This constitutes a significant limitation in our research design, which we have explicitly acknowledged in the revised manuscript by: 1) adding a statement about "lack of direct attention measurement" in the methodological limitations section, and 2) modifying all inferential statements about attention mechanisms in the Discussion to incorporate cautious terminology like "potentially" and "speculatively." Your professional critique has helped us more rigorously align theoretical explanations with empirical evidence, which is fundamentally important for enhancing the quality of our research.

Although this study did not employ eye-tracking or subjective attention scales to directly record learners’ visual attention distribution, the signaling effect documented in analyses of multimedia learning and related empirical studies supports the effectiveness of marker cues in guiding attention. For example, Zhang et al. demonstrated that overlaying expert gaze trajectories on videos effectively guided learners to key areas and optimized visual search patterns [55]. Similarly, soccer observational learning research revealed that combined visual and verbal cues significantly reduced movement errors and enhanced performance. On the basis of this evidence and the enhanced motor learning outcomes observed in the present study, this study hypothesized that visual marker cues likely enhanced learning by directing learners’ visual attention to critical movement components. However, the potential roles of other attention-guiding mechanisms in this process warrant further study [56].（Lines：659-669）

Supplementary references：

55. Zhang W, Wang Y, Feng Z, Zhu S, Cui J, Hao W, Wang C. A method to improve the hazard perception of young novice drivers based on Bandura’s observational learning theory: Supplement to expert commentary training. Transp Res Part F Traffic Psychol Behav. 2022;85:133-149. doi:0.1016/j.trf.2022.01.005

56. Hodges NJ, Williams AM, Hayes SJ, Breslin G. What is modelled during observational learning? J Sports Sci. 2007;25(5): 531–545. doi:10.1080/02640410600946860

**8 Minor Revisions:**

Minor revisions are recommended to improve grammar, sentence structure, and overall flow, as several sections contain typographical errors, awkward phrasing, and inconsistent formatting that could hinder readability. See some examples below:

8.1. Line 34: Verb tense should say ‘enables’

8.2. Line 164: missing space after the period

8.3. Line 212: Sentence is the past tense.

8.4. Line 455 – sentence ends with an unnecessary ‘is’

8.5. Line 511 – likely a typo ‘studing’ – ‘studying’

We sincerely appreciate your meticulous review of the manuscript's language quality! These observations made us acutely aware of our shortcomings, and we have systematically addressed all identified grammatical, syntactic, and formatting issues, specifically including:

8.1 In the Introduction section of academic papers, when describing general facts, theoretical foundations, or established principles, the Simple Present Tense should be used. As non-native English speakers, to the best of our limited English proficiency, we have ensured constructions like "Vision enables" maintain the Simple Present Tense.

Vision **enables** learners to engage in observational learning, and hearing enables them to receive verbal instructions [7-9]. （Lines：50-51）

8.2 We have added spaces after all punctuation marks throughout the manuscript.（Lines：234）

8.3 We have corrected the tense consistency. Thank you.

The experiment **took** place from September 9th, 2024 to September 27th, 2024. （Lines：350-351）

8.4 We have removed the unnecessary "is" at the end of sentences.

Studies have reported that combining visual information with auditory instructions is more effective than combining visual information with textual instructions [6,58].（Lines：696-698）

8.5 We have corrected the spelling error, changing "studing" to "studying".

For example, in teaching set shots, using video with narration and markers (guided dual-sensory information) during the preparation stage can assist with **studying** upper-limb movement techniques, whereas video with narration (dual-sensory information) can assist with the execution stage motor learning. （Lines：787）

**Reviewer 2**

**Abstract**

Line 2 : “for enhancing”… or “to enhance”

Based on your suggestion, we have revised 'for enhancement' to 'to enhance'.

The effectiveness of instructional information is crucial to enhancing motor learning outcomes.（Lin：2-3）

Line 5 : The concept of 'Movement Staging' is not very clear. It would be helpful to provide a more detailed explanation or clarification on what exactly this term refers to and how it is applied in this context.

Thank you sincerely for your valuable suggestions on the concept of "action staging"! We fully agree that this term requires a more precise academic definition. Based on your guidance, we have supplemented the definition explanations from three dimensions: theoretical origins (Fitts three-stage model), empirical evidence (Wulf et al.'s research), and teaching practice. Special emphasis: 1) Added Magill's (2010) authoritative definition to clarify conceptual boundaries; 2) Using Schmidt schema theory to explain the shaping effect of stages on action representation; 3) Supplement the empirical evidence of cognitive load by Guadagnoli (2004). These modifications not only improve the terminology explanation, but also highlight the key value of staged teaching in optimizing beginners' cognition in this study. Your professional opinion greatly enhances the theoretical depth of the paper, and we deeply appreciate it.

Movement staging refers to dividing motor skills into consecutive logical phases on the basis of their temporal sequence and technical structure to facilitate systematic learning, training, and correction [38]. Movement staging is a fundamental instructional, training, and analytical tool in motor learning. Fitts’ three-stage model of movement staging [39] posits that decomposing movements to reduce cognitive load is critical to skill acquisition. Additionally, Schmidt’s schema theory suggests that phased practice establishes refined motor schemata and enhances movement adaptability [40]. Studies have verified that step-by-step practice reduces cognitive load in beginners, optimizes learning efficiency, and accelerates error correction through phased feedback [41,42]. However, movement staging is not a fixed, inflexible process; instructors may divide phases according to instructional needs, constructing cognitive frameworks through phase decomposition to enhance learning efficiency.（Lin：140-151）

Supplementary references：

38. Magill RA, Anderson DI . Motor learning and control. New York, NY: McGraw-Hill Publishing; 2010.

39. Fitts PM. Human performance. Brooks/Cole Press; 1967.

40. Schmidt RA. A schema theory of discrete motor skill learning. Psychol Rev. 1975;82(4): 225–260. doi: 10.1037/h0076770

41. Wulf G, Shea CH. Principles derived from the study of simple skills do not generalize to complex skill learning. Psychon Bull Rev. 2002;9.2:185-211. doi: 10.3758/BF03196276

42. Guadagnoli MA, Lee TD. Challenge point: A framework for conceptualizing the effects of various practice conditions in motor learning. J Mot Behav. 2004;36(2):212-224. doi:10.3200/JMBR.36.2.212-224

Line 7: "3" should be written as "three" in formal writing.

Line 9: "3" should be written as "three" in formal writing.

We are ashamed that you pointed out such an error and have corrected it.

A teaching experiment involving 132 middle school students was used to analyze **three** instructional methods: visual-only (video), visual–auditory (video with narration), and guided dual-sensory information with visual cues (video with narration and markers). Participants learned set shooting over **three** weeks.（Lin：6-9）

Line 11: Use "In terms of timing” to replace “temporally”

Line 13 "Spatially, upper-limb motor learning required more guidance than lower-limb motor learning."

Thank you for your suggestion, as the error in the results section of the revised abstract no longer exists.

Inferential statistics must be reported in the results section.

Thank you for the expert reviewer's correction. We have added statistical inference to the "Results" section and organized the structure of the connection between the two.

The results revealed that (1) audiovisual (dual-sensory) information considerably enhanced movement pattern performance, and dual-sensory information with additional visual markers strengthened movement pattern retention and optimized upper-limb movement pattern performance during the preparation stage. (2) All three groups (visual-only, dual-sensory, and dual-sensory with visual markers) exhibited improved movement result performance after training, with the group receiving guided dual-sensory instruction exhibiting substantially superior posttest movement result performance than the group receiving video alone. (3) Both the group receiving video with narration and the group receiving guided dual-sensory instruction exhibited substantial improvements in motor cognition, with the dual-sensory information with visual cues associated with the strongest facilitation effect on upper-limb cognition during the execution stage. These results reveal that guided dual-sensory information substantially enhances motor learning outcomes in basketball shooting, with required movement guidance of the upper-limb segments being greater than that for other body segments.（Lin：10-23）

**Introduction**

The introduction presents the context well in terms of theoretical and empirical approaches to motor learning. a few clarifications and modifications are required

Line 26 : Can you illustrate the spatiotemporal dimension of motion?

Thank you for your detailed suggestions and for helping me annotate each line. I have added an explanation of the temporal and spatial aspects of the action. Your meticulous research attitude inspires me to actively learn.

A self-developed scale called the Set Shot Movement Pattern Evaluation Scale was used to evaluate movement pattern performance in upper-limb movement pattern, lower-limb movement pattern, and ball-handling movement pattern during the preparation and execution stages of a set shot. The preparation stage spans ball reception to movement initiation, whereas the execution stage extends from movement initiation until ball release [44,45].（Lin：322-327）

Line 28 : The research question should not appear here, but rather at the end of the presentation of your theoretical elements

Thank you for your revision suggestion. We have moved the research question to after the theoretical elements have been stated.

The recognition of these deficiencies in set shot pedagogy prompted the following research question: Which of the three information presentation methods—visual-only (video), dual-sensory audiovisual (video with narration), and guided dual-sensory instruction (video with narration and markers)—is most effective in motor learning?（Lin：182-185）

Line 35: present the role of information in motor learning. What are they used for and when? See perhaps the work on internal models ...

Sincerely thank you for your profound insight into the information processing mechanism. We have made the following improvements to the relevant content based on internal model theory: 1) Clarify the specific functions of information in each stage of action learning, including input in the perceptual stage, integration in the decision-making stage, and regulation in the execution stage; 2) Provide additional explanation on how visual and auditory information affect the establishment and updating of internal models through different neural pathways; 3) Emphasize the close relationship between error detection capability and information feedback, especially the crucial role of proprioceptive information in action correction. We have added relevant content in the discussion section of the paper, and provided examples in the next response.These modifications make the explanation of information processing mechanisms more systematic and complete. Your professional guidance has made the mechanism of this research more explanatory, for which we are deeply grateful.

Information is key in motor learning because perception and movement influence each other. Most sensory information for skilled actions is obtained from the environment, primarily through vision and hearing. Vision enables learners to engage in observational learning, and hearing enables them to receive verbal instructions [7-9]. （Lin：49-51）

Motor skill acquisition follows a staged information processing flow comprising stimulus identification, response selection, and response programming. In the perceptual stage, learners receive feedback regarding environmental and bodily states through sensory channels that enables them to identify action goals and contextual cues. During the decision stage, learners retrieve movement schemas from long-term memory on the basis of perceptual input to determine and plan motor responses. The execution stage involves translating motor plans into neuromuscular commands to execute actions [38]. Because information input occurs during stimulus identification in the perceptual stage, learners must identify key information before interpreting its attributes.（Lin：625-633）

Supplementary references：

38. Magill RA, Anderson DI . Motor learning and control. New York, NY: McGraw-Hill Publishing; 2010.

Line 36 : Can you provide an example of the importance of external (and internal) information in the execution of movements and in learning, based on the literature?

Thank you for the expert's correction. We have added a classic example: in primary school long jump teaching, the video observation group significantly improved the action effect through external visual cues (such as landing area markers), which activates the mirror nervous system to reduce cognitive load. This example further confirms the optimization effect of external information on perception action coupling.

For example, one study reported that video observation considerably enhanced elementary school students’ long jump skill motor learning outcomes [2]. Video observation employs external information to guide learners to focus on their movements, activating the mirror neuron system to achieve automated “observation to imitation”and reduce the cognitive load required to maintain internal control. Such external visual cues efficiently promote the acquisition and transfer of motor skills by enhancing perception–action coupling.（Lin：61-67）

**Method**

The method needs to be rewritten as it is currently unclear. It should be reorganized following this outline:

Population : Add gender distribution

Thank you for your suggestions on the details of this study. The basic information of the participants is very important, and we will carefully supplement the relevant content. The gender distribution has been supplemented, and the number of male and female students in the class is basically the same. In this experiment, under the premise of controlling the consistency of teaching process and content, the gender indicators of the subjects were not included in the measurement variables. These supplementary contents have been reflected in the research object section of the revised manuscript. We sincerely thank you for pointing out this key methodological issue, which significantly enhances the rigor of the research.

The participants were randomly assigned to one of three instructional conditions: a video group (n = 44), a video with narration (audiovisual) group (n = 44), and a video with narration and markers (guided dual-sensory instruction) group (n = 44), with Classes A (video) and B (video with narration) each contributing 22 male and 22 female students, and Class C (video with narration and markers) contributing 21 male and 23 female students.（Lin：213-218）

Experimental Design: Specify the three conditions... Line 163-164: it is not clear.

Materials: Describe what is common between the three videos as well as what distinguishes them. What is shown in each video? What instructions are provided? What are the markers? What is explained from line 172 to 179 should appear in this paragraph.

Thank you for your prompt. After considering the above two issues together, we have explained the teaching conditions in the research tool - learning materials. The following is the content we have added.

The video group was exposed to a set shot skill acquisition video. The information was delivered through a visual only sensory modality consisting exclusively of basketball shooting demonstration footage.

The audiovisual (video with narration) group received the same video content as the visual-only group but with the addition of voice-over narration. The video presented basketball shooting demonstration footage synchronized with verbal explanations of movement essentials. The audio content precisely matched the key movement components and was temporally aligned with the video presentation.

The group receiving video with narration and markers received the previous two information types plus additional visual markers. Research has demonstrated that incorporating guiding information considerably enhances retention test performance and increases learners’ fixation duration toward and frequency of fixating on marked cue areas [47]. Additionally, research has indicated that physical marker cues (e.g., flashing arrows) in key information areas effectively guide attention [48]. On the basis of these findings, the guided dual-sensory video materials in this study incorporated localized markers targeting movement essentials, which appeared on corresponding limb segment displays without obscuring critical movement feature areas. The marking method employed low-saturation red flashing frames that appeared synchronously with movement essentials presented through both video and narration. Each marker was presented for 1 second and flashed only once before transitioning to a static red frame. Preliminary experimental results on changes in learning outcomes suggested that the presentation of the markers did not induce unintended visual bias, distract attention from critical movement features, or give rise to redundancy effects.

The filming protocol was established on the basis of the temporal sequence of set shot execution, incorporating the recommendations of expert consultations and referencing the basketball textbooks edited by Sun Minzhi in addition to findings in the literature [44,45]. The learning information provided in the video materials was consistent with both the Set Shot Movement Pattern Evaluation Scale and the Set Shot Motor Cognition Test, establishing alignment across learning materials, motor skill assessments, and motor cognition evaluations.（Lin：266-295）

Procedure: “Participants were randomly assigned to one of the three groups. Each participant watched a video corresponding to their group and then completed a motor cognition and performance test…” Include details such as the number of sessions, duration, etc . Explain which evaluation scales were taken and at what time.

We sincerely appreciate the expert's corrections! We have supplemented the experimental procedure details: a total of 6 sessions over 3 weeks, with clearly defined time points for pre-test (Session 1), post-test (after Session 6), and retention test (48 hours later), specifying the use of assessment tools like the "Set Shot Movement Pattern Evaluation Scale" and their administration timelines, while standardizing the warm-up, video viewing, and practice protocols.

The main experiment involved the participants engaging in 3 weeks of learning, with two sessions per week (The experiment took place from September 9th, 2024 to September 27th, 2024.).

All three participant groups were instructed by the same physical education teacher; two assistants (graduate students in sports science) assisted with data collection. The procedures of the experimental phase were as follows. During the first session, pretest data on set shot movement result performance were collected by evaluating set shot movement pattern ratings (Set Shot Movement Pattern Evaluation Scale). Sessions 2–6 focused on motor skill acquisition, with each session comprising the following standardized procedures: (1) group warm-up exercises for all participants in the same condition group led by the physical education teacher (7–8 minutes); (2) teacher-directed, condition-specific instructional video viewing (repeated 3 times, 4–5 minutes); (3) guided practice with outcome-based feedback (teacher comments on movement accuracy alone without making corrections), maintaining equal feedback frequency across classes (two instances per participant; 10–12 minutes); (4) repeat video viewing (4–5 minutes); (5) repeat guided practice (10–12 minutes); (6) instruction to avoid practice outside of instructional hours. Immediately after the conclusion of the sixth session, assessments of set shot motor cognition (Set Shot Motor Cognition Test), set shot movement result performance, and set shot movement pattern ratings (Set Shot Movement Pattern Evaluation Scale) were administered. A retention test was administered 48 hours after the posttest (Figure 1).（Lin：350-369）

A diagram with evaluation and intervention to illustrate the procedure would be helpful.

Your suggestion is very good and has been of great help to our research. We have created a new diagram in the experimental process to help readers understand the process of teaching and evaluation.

Line 163 : the same sentence twice.

Thank you for your careful review. What I want to express here is the combination of multimedia and sensory information. The wording is slightly the same, but the latter sentence mainly expresses the message.

Line 215: Feedback – What information is provided?

Thank you for the expert's correction. We have clarified the nature of the feedback: teachers only provide standardized outcome evaluations (such as "correct/incorrect shooting movements"), and guidance on correcting movements is strictly prohibited throughout the process to ensure consistency in the content and frequency of the three sets of feedback.

(3) guided practice with outcome-based feedback (teacher comments on movement accuracy alone without making corrections), maintaining equal feedback frequency across classes (two instances per participant; 10–12 minutes);（Lin：361-363）

**Learning outcome data collection**

Line 221 : add after motor cognition “(Set Shot Motor Cognition Test)”

Thank you for helping us improve the title. The "Learning outcome data collection" section mainly explains how to collect data, and the detailed description of the tool is already reflected in the "Research instruments" section. At the same time, your suggestion to clarify the tools is very important, as we have provided explanations in each 'Research instruments' section. Thank you again for your help.

Motor cognition was assessed using the self-developed Set Shot Motor Cognition Test, which comprised 18 multichoice questions. （Lin：375-376）

Line 222: Set Shot Motor Cognition Test – Did you create this scale? If so, could you provide examples of the items? Who completes it?

Thank you for the expert's inquiry. This' Set Shot Motor Cognition Test 'is a scale we have independently developed, and the validity of the test paper is based on triple validation: content validity: the questions are strictly anchored to the core concepts of the' Fixed Point Shooting Action Type Evaluation Scale ', and have been reviewed and revised by four domain experts (one frontline teacher and three basketball experts); Structural validity: The pre-test uses difficulty analysis and discriminant optimization to ensure that the questions can effectively distinguish the level of action understanding. Reliability guarantee: Half fold reliability alpha=. 82 meets the psychometric standard (>. 70). After removing the unqualified items from the final scale, all indicators met the measurement standards. This study ensures the unity of the toolchain through a triple design: the three tools are actually operational closed loops of the same action concept, and their consistency is not a subjective assertion, but is supported by both design logic and empirical data. We are adding content to the research tools section. Thank you for your prompt, which has made our research methodology more comprehensive.

The study assessed set shot motor cognition by using the Set Shot Motor Cognition Test, a multichoice questionnaire based on the Set Shot Movement Pattern Evaluation Scale. The test content aligned with the key movement points and essential elements highlighted in the instructional video.An initial draft of the test was reviewed by basketball professionals, and the draft was refined on the basis of their feedback. A pilot test was then conducted. In total, 50 valid questionnaire responses were received in the pilot test. The discrimination index (D) indicates item discriminatory ability and reliability, with higher values indicating greater discriminatory ability and reliability. Items with D values < 0.2 should be eliminated, and items with D > 0.4 have high discriminatory ability and reliability and should be retained. The difficulty index (P) reflects item difficulty, with higher values indicating lower difficulty; the acceptable range is from .20 to.80, with the optimal mean of .50 [49]. To enhance test validity, this study conducted a distractor analysis after the pilot test. Any distractors selected significantly more or less frequently than anticipated or chosen by less than 5% of the examinees were eliminated in subsequent refinements to the test. The Set Shot Motor Cognition Test was finalized by adjusting both the frequency and sequence of correct answers to ensure balanced distribution and randomization across items.（Lin：298-313）

Line235: add after movement pattern performance “(Set Shot Movement Pattern Rating Scale”)

Thank you for helping us improve the title. The "Learning outcome data collection" section mainly explains how to collect data, and the detailed description of the tool is already reflected in the "Research instruments" section. At the same time, your suggestion to clarify the tools is very important, as we have provided explanations in each 'Research instruments' section. Thank you again for your help.

Three experts evaluated movement pattern performance by using the Set Shot Movement Pattern Evaluation Scale. （Lin：389-390）

PS: "Set Shot Movement Pattern Rating Scale" has been unified as "Set Shot Movement Pattern Evaluation Scale" throughout the text.

Line 236: Set Shot Movement Pattern Rating Scale – Where does this scale come from?

We sincerely appreciate the expert's attention. This study ensures instrumentation coherence through a triple-design approach: the three tools form an operationalized closed-loop measuring the same motor construct, with their consistency substantiated by both design logic and empirical data rather than subjective claims. The set shot multimedia learning materials, movement pattern evaluation scale, and cognitive test were all rigorously developed based on the same movement analysis model (structured according to spatiotemporal dimensions of movement staging and limb segments, categorizing elements as upper-limb movement pattern performance, lower-limb movement pattern performance, and ball-control performance during preparation and execution stages). We have supplemented the research instruments section accordingly - your insightful suggestion has significantly improved our methodological reporting.

The learning information provided in the video materials was consistent with both the Set Shot Movement Pattern Evaluation Scale and the Set Shot Motor Cognition Test, establishing alignment across learning materials, motor skill assessments, and motor cognition evaluations.（Lin：292-295）

The internal consistency of the Set Shot Movement Pattern Evaluation Scale items was determined on the basis of the pilot test data. On the basis of the valid sample (N = 50), the total scale had a Cronbach’s α coefficient of 0.88 [95% confidence interval: 0.86, 0.90], indicating excellent internal consistency [49].（Lin：328-331）

Line 245: Interrater reliability – Which reference are you relying on to justify that the reliability is good?

Thank you for the reviewer's correction. The determination of inter/intra rater consistency strictly follows the action measurement standard proposed by Williams&Lacy (2018): "Kendall's W>0.75 is considered excellent reliability and requires significant chi square (p<. 05)." In this study, the upper limb (W=0.89), lower limb (W=0.90), ball control (W=0.94), and test-retest reliability (W=0.93-0.97) all exceeded this threshold, with significant chi square values (ps<. 001), fully meeting the "good consistency" standard of this authoritative literature. We are providing additional content explanations in the research tools section. Thank you for your prompt.

The internal consistency of the Set Shot Movement Pattern Evaluation Scale items was determined on the basis of the pilot test data. On the basis of the valid sample (N = 50), the total scale had a Cronbach’s α coefficient of 0.88 [95% confidence interval: 0.86, 0.90], indicating excellent internal consistency [49].

Interrater reliability was assessed using videos of the first 10 participants and evaluated using Kendall’s coefficient of concordance. The coefficients for upper-limb, lower-limb, and ball-handling movement patterns were 0.89, 0.90, and 0.94, respectively, with χ2 values of 21.20, 22.68, and 24.21, respectively; all values indicated significant concordance, implying good interrater reliability.

Internal reliability of raters was also assessed using videos of the top 10 participants, with 2 assessments being conducted 2 weeks apart to check test–retest reliability. Kendall’s coefficient of concordance revealed high reliability, with coefficients of 0.93, 0.94, and 0.97 for upper-limb, lower-limb, and ball-handling movement patterns, respectively, and corresponding χ2 values of 25.10, 26.34, and 27.71. All values indicated significant consistency.（Lin：328-342）

Supplementary references：

49. Williams SM, Lacy A. Measurement and evaluation in physical education and exercise science. Routledge, 2018.

**Data analyses**

If I understand correctly, you performed an ANOVA at baseline and then another ANOVA at post-intervention. Why did you not conduct a one-way ANOVA with repeated measures and a factor of group: TIME (pretest vs posttest vs retention) x GROUP (video vs video with narration vs video with narration and markers)? It seems important, when discussing learning, to include a comparison between the data before and after the intervention (in addition to the comparison between groups).

Thank you for your suggestion. With reference to your suggestions, we have revised our research method and re analyzed the differences in the performance of fixed-point shooting action types and fixed-point shooting results. We have also written the results section.

A mixed-design ANOVA was employed to examine differences in set shot movement pattern performance and set shot outcome performance across the three information presentation groups (video, video with narration, and video with narration and markers) between the pretest and posttest. When significant group × time interactions were observed, simple main effects analyses were conducted, followed by post hoc comparisons using the least significant difference (LSD) method (α = .05). When nonsignificant interactions were identified, the main effects were analyzed separately using LSD post hoc tests (α = .05). (Lin：412-415）

Most of the content in the Results section（Lines：417-589）

It seems important, when discussing learning, to include a comparison between the data before and after the intervention (in addition to the comparison between groups).

Sincerely thank the expert reviewers for their insightful guidance. Your core suggestion on "comparing data before and after intervention" has made us deeply aware of the crucial role of longitudinal analysis in interpreting learning outcomes. We have comprehensively revised the discussion section:

First, regarding movement pattern performance, a comparison before and after multimedia instruction revealed that visual–auditory yielded the best motor skills retention results. Despite some studies have suggested that multimedia instruction does not affect motor learning skills [20,30,31]; however, in the present study, set shot movement pattern performance significantly improved in the video with narration and video with narration and markers groups. (Lin：602-607）

Second, pre–post instructional comparisons of movement result performance revealed considerable improvements in shooting accuracy across all three groups, with both posttest and retention test scores exceeding the scores on the pretest. When comparing movement pattern performance and movement result performance, the temporal trends in learning gains were consistent—regardless of information presentation method, six instructional sessions induced positive changes in set shot movement patterns that subsequently translated to enhanced movement results. (Lin：686-692）

Nevertheless, the retention test results revealed no significant differences in shooting accuracy (movement result performance) among the three groups, primarily because the group receiving guided dual-sensory information group exhibited substantial performance reductions over time. Although dual-sensory guidance temporarily enhanced movement pattern performance, its learning effects may be unstable and exhibit noticeable performance decay during retention testing. This finding suggests that the intervention may have facilitated only the shallow encoding of motor skills. This finding aligns with those of a study [54] indicating that neither multimedia presentation method nor measurement phase substantially enhanced shooting accuracy retention.（Lin：699-707）

**Results :**

Please report the effect sizes.

Additional explanation of effect sizes has been provided.

It would seem more appropriate to report all the effects in the text and illustrate

Thank you for your suggestion. All results have been presented in text. As this experiment is based on spatiotemporal dimension analysis, there are too many factors related to action divergence and movement segments, so this section is mostly described in text.

Line 288 replace “=0.00” by “p<0.001”

Thank you once again for your patience and meticulousness. I have learned a lot from your review, and this serious spirit is the cornerstone of academic research. You have set an example for me. All significant values in this article have been uniformly set to. 05, and values below this level are marked as p<. 05.

**Tables**

The tables are not presented in the same way, and the groups are not always named consistently . Please standardize them (If you choose to keep the tables).

Thank you sincerely for your diligent and meticulous review. I want to learn from your research spirit. Based on the suggestions in section 5.1, I have redesigned the statistical method, and the data presentation of fixed-point shooting action forms and fixed-point shooting results has also been changed, without retaining the original table. Please refer to the Results section of the paper for details.

The post-hoc statistics are not reported (they should be included in the text).

The post hoc statistical results have been presented in the results section of the paper in written form. Thank you for your careful review.

**Discussion**

The discussion is generally clear but it would benefit add more connections between results of this study and some studies suggest contradictory findings:

Your question prompted us not only to clarify our own findings, but also to connect and compare our research results more profoundly with the opposite results in previous studies, providing a key explanation for the differences in results and significantly enhancing the academic depth and persuasiveness of the research. The following is a supplementary explanation for your insights:

Despite some studies have suggested that multimedia instruction does not affect motor learning skills [20,30,31]; however, in the present study, set shot movement pattern performance significantly improved in the video with narration and video with narration and markers groups. Guided dual-sensory information aligns with both the dual-channel principle and the signaling principle of CLT [11].（Lin：604-608）

Nevertheless, the retention test results revealed no significant differences in shooting accuracy (movement result performance) among the three groups, primarily because the group receiving guided dual-sensory information group exhibited substantial performance reductions over time. Although dual-sensory guidance temporarily enhanced movement pattern performance, its learning effects may be unstable and exhibit noticeable performance decay during retention testing. This finding suggests that the intervention may have facilitated only the shallow encoding of motor skills. This finding aligns with those of a study [54] indicating that neither multimedia presentation method nor measurement phase substantially enhanced shooting accuracy retention.（Lin：699-707）

The participants were engaged in the earliest stages of shooting learning, in which movement execution is slow and unsteady and improved movement patterns do not translate to increased shooting accuracy, reflecting a form–function dissociation in complex motor skills that is consistent with the predictions of the hierarchical skill acquisition model [39]. More importantly, movement result performance alone cannot evaluate the temporal and spatial process of learners’ movements. Outcome performance alone does not fully reflect the effectiveness of the learning process because it does not capture the timing and spatial aspects of movements. This may explain why findings have often varied in motor learning studies.（Lin：722-730）

It would be beneficial to explicitly address why these discrepancies exist—such as differences in methodology, participant characteristics (age), or the type of skills being learned.

In the discussion section, we analyzed the reasons for the differences in results from the following perspectives

Motor learning is the process through which individuals achieve persistent changes in motor skills through accumulated practice and experience [4]. Although the guided dual-sensory learning group exhibited considerably superior movement pattern performance than the visual-only group, this advantage did not consistently translate into greater shooting accuracy. This result indicates that although information presentation substantially influenced movement pattern learning, shooting accuracy—a higher-order motor skill —likely requires multisystem integration and thus demands more prolonged, specialized practice to achieve substantial improvements in precision.（Lin：708-715）

Regarding motor cognition, no significant differences were observed in lower-limb cognition scores among the three groups during either the preparation or execution stages, a result consistent with findings in the literature on movement pattern performance. This result suggests a correlation between motor cognition and motor performance and reflects the requirement to consider cognitive differences in movement staging and motor sequencing when complex technical skills are taught through multimedia, particularly when the learned actions involve multiple limb segments.（Lin：761-767）

The interpretation of results could be clarified. For instance, when mentioning that adding markers "did not create a redundancy effect," can you explain this reported redundancy effects with multimedia learning.

Thank you sincerely for your profound insights and valuable opinions! Your precise question about the "redundancy effect" not only highlights the core concept of multimedia learning theory, but also guides us to reflect and deepen our interpretation of the research findings. Your question is highly constructive and has significantly improved the clarity and persuasiveness of the theoretical mechanism explanation in our paper, making the core argument of "labeling guides attention without triggering redundancy" more solid and powerful. The following is a detailed supplementary explanation for your valuable feedback:

The superior learning outcomes of the group receiving guided dual-sensory instruction suggest that these learners did not experience redundancy effects despite the increased information load during basketball shooting training. Research employing different multimedia presentation formats to teach shooting skills revealed that video and on-screen text produced the least optimal results in movement integration instruction. The researchers attributed this result to competition between two types of visual stimuli, particularly to the redundant information provided through text (i.e., the redundancy effect) [54]. However, when information resources are properly allocated, learning results can be optimized. In the current study, for basketball shooting, adding marker information did not create a redundancy effect; rather, it provided attentional guidance, resulting in superior movement patterns and shooting skills [50]. The redundancy effect involves a “cognitive efficiency penalty caused by information repetition” [5]. In this study, the video (visual stimulus) showed the relevant techniques in their entirety, the narration (auditory stimulus) explained the principles underlying the movements, and the markers (visual cues) directed the learner’s attention to key areas; these three elements complemented one another rather than introducing redundancy effects. This finding demonstrates that in motor skill instruction, when multimodal information elements perform distinct roles, they can mitigate redundancy and lead to the optimal allocation of cognitive resources.（Lin：642-659）

The statement that "markers highlight key details, which assist learners in establishing memory schemas and improves error detection" is interesting, but it would benefit from additional supporting evidence or references to studies that have shown these specific cognitive processes in motor learning.

Your valuable feedback is the key to enhancing the theoretical rigor of this study, prompting us to provide a detailed explanation of the information processing stage (perception decision execution), thus more systematically demonstrating how labeling affects the starting point of cognitive processes. The following are specific supplements and revisions based on your insights:

Motor skill acquisition follows a staged information processing flow comprising stimulus identification, response selection, and response programming. In the perceptual stage, learners receive feedback regarding environmental and bodily states through sensory channels that enables them to identify action goals and contextual cues. During the decision stage, learners retrieve movement schemas from long-term memory on the basis of perceptual input to determine and plan motor responses. The execution stage involves translating motor plans into neuromuscular commands to execute actions [38]. Because information input occurs during stimulus identification in the perceptual stage, learners must identify key information before interpreting its attributes.（Lin：625-633）

It might be relevant to discuss the fact that in this study these are novices. The results may be different for experts ....

Thank you for your question. The participants in this study are beginners. If the subjects were experts, would the results be different? This is a very thought-provoking research point, and I will write this idea in future research.

This study has several limitations. The study included a limited number of middle school students; therefore, the results should be generalized with caution. Additionally, the findings indicating that marker cues enhance learning outcomes through attention guidance mechanisms were derived indirectly in the absence of direct attentional measurement. Future research should explore different learning content and include a larger, more diverse cohort of learners across various sports disciplines. Future studies should incorporate expert learners to examine the differential effects of information presentation on individuals who have already acquired fundamental motor skills.（Lin：794-801）
