# Supplementary material for: Effect of guided dual-sensory information on motor learning outcomes based on spatiotemporal dimensions
Source: PLoS One. 2025 Nov 20;20(11):e0337236. doi: 10.1371/journal.pone.0337236 (PMC12633867; doi:10.1371/journal.pone.0337236)
Supplement: Attachment — Submitted filename: reviewing_2.docx [file pone.0337236.s004.docx]

This revised version shows clear progress compared to the previous one. The presentation of the methodology is now much clearer, the statistical analyses are more rigorously applied, and the overall discussion appears more coherent and well-structured.

**Abstract**

Line 25 : It would seem more appropriate to place point (2) before point (1), moving from the more general to the more specific, and to avoid repeating the group comparison.

**Method**

Participants :

You do not specify how the randomization was carried out

Line 240 and 247 : You repeat « consent forms were signed by the participants’ parents »

Line 259 : Specify why the 0.8 threshold is chosen as a reference (often attributed to Cohen, 1988), as this would strengthen the justification.

Line 272 « Movement patterns and movement performance were assessed using a mixed-design **ANOVA**, … »

The 3 conditions are much better presented , which makes it easier for the reader to understand.

Line 320 : Could you justify the thresholds used (0.2; 0.4...)?

**Figure**

Could you make three squares of the same size for the three conditions and explain in the legend that each participant only completed one of the three conditions? For the time labels, sometimes the first letter is capitalized and sometimes not; please harmonize. It would be more appropriate if the retention test followed the post-test in a linear way (by enlarging the figure and continuing on the next page). The phrase “After 6 classes” is misplaced, as it gives the impression that six classes occur between the end of the protocol and the posttest session.

Learning outcome data collection

Line 389 and….: Could you provide examples of items or questions for each of the scales (motor cognition, movement result performance...)?

**Results :**

It would seem more appropriate to present the results in the form of graphs rather than tables.

**Discussion :**

Line 665-672 : Be careful in your interpretation, this remains a hypothesis.

Line 673-683 : The authors argue that visual cues enhance learning by guiding attention. Given that no direct measures of attention (e.g., eye-tracking, subjective scales) were used, this limits the robustness of the interpretations. I suggest qualifying the conclusions on this point and stating more explicitly that these results remain hypothetical.

Line 676 : « For example, Zhang et al. » the date is missing after the reference

Line 649 : You discuss multimodality as the contribution of complementary information, but in itself multimodality can improve motor performance and learning, as seen in these papers :

- Blais, M., Jucla, M., Maziero, S., Albaret, J. M., Chaix, Y., & Tallet, J. (2021). Specific cues can improve procedural learning and retention in developmental coordination disorder and/or developmental dyslexia. *Frontiers in Human Neuroscience*, *15*, 744562.
- Lagarrigue, Y., Cappe, C., & Tallet, J. (2021). Regular rhythmic and audio-visual stimulations enhance procedural learning of a perceptual-motor sequence in healthy adults: A pilot study. *PLoS One*, *16*(11), e0259081.
- Diederich A, Colonius H. Bimodal and trimodal multisensory enhancement: Effects of stimulus onset and intensity on reaction time. Perception & Psychophysics. 2004 Nov;66(8):1388–404. pmid:15813202
- Hecht D, Reiner M, Karni A. Multisensory enhancement: gains in choice and in simple response times. Exp Brain Res. 2008 May 14;189(2):133. pmid:18478210
