# Supplementary material for: Effect of guided dual-sensory information on motor learning outcomes based on spatiotemporal dimensions
Source: PLoS One. 2025 Nov 20;20(11):e0337236. doi: 10.1371/journal.pone.0337236 (PMC12633867; doi:10.1371/journal.pone.0337236)
Supplement: Attachment — Submitted filename: R2 Response to Reviewers 20251023.docx [file pone.0337236.s006.docx]

Dear Editor

Thank you for your insightful suggestions. Our team has carefully studied each of the revision suggestions for this article and made corresponding modifications. We hope that our responses address the reviewers' comments satisfactorily, and we would be pleased to consider any further suggestions. Thank you for your thorough review of this article once again.

Best regards,

The authors

The following is a revision explanation:

| • Black text | represents the original comments from the reviewers |
| --- | --- |
| • Purple text | indicates our direct responses to the reviewers' comments |
| • Blue text | shows the revised content that corresponds to changes made in the revised manuscript |

**Reviewer 1**

**Abstract**

**Line 25 : It would seem more appropriate to place point (2) before point (1), moving from the more general to the more specific, and to avoid repeating the group comparison.**

Thank you for your suggestion and for reading this article so carefully. The correction has been completed.

(1) All three groups (visual-only, dual-sensory, and dual-sensory with visual markers) exhibited improved movement result performance after training, with the group receiving guided dual-sensory instruction exhibiting substantially superior posttest movement result performance than the group receiving video alone. (2) audiovisual (dual-sensory) information considerably enhanced movement pattern performance, and dual-sensory information with additional visual markers strengthened movement pattern retention and optimized upper-limb movement pattern performance during the preparation stage. （Lines：25-31）

**Method**

**Participants :**

**You do not specify how the randomization was carried out**

The study school admits students using a proximity-based enrollment policy and maintains regular mixed-gender classes. Under these conditions, the motor abilities of the students in the same grade approximate a normal distribution, satisfying random sampling criteria.

We employed cluster sampling, randomly selecting three out of the nine seventh-grade classes. （Lines：227-228）

**Line 240 and 247 : You repeat « consent forms were signed by the participants’ parents »**

The second occurrence of « consent forms were signed by the participants’ parents » has been deleted. Thank you for your careful review.

**Line 259 : Specify why the 0.8 threshold is chosen as a reference (often attributed to Cohen, 1988), as this would strengthen the justification.**

Relevant literature support has been added.

The achieved power of 0.87 met the 0.8 threshold [46]. （Lines：258）

46. Cohen J. Set correlation and contingency tables. Applied psychological measurement. 1988; 12(4): 425-434.

**Line 272 « Movement patterns and movement performance were assessed using a mixed-design ANOVA, … »**

**The 3 conditions are much better presented , which makes it easier for the reader to understand.**

Thank you again for the meticulous correction. It has been completed.

Movement patterns and movement performance were assessed using a mixed design ANOVA, with……（Lines：271-272）

**Line 320 : Could you justify the thresholds used (0.2; 0.4...)?**

Supplementary references have been provided to explain the applicable thresholds.

Items with D values < 0.2 should be eliminated, and items with D > 0.4 have high discriminatory ability and reliability and should be retained[49]. （Lines：391-320）

**Figure**

**Could you make three squares of the same size for the three conditions and explain in the legend that each participant only completed one of the three conditions? For the time labels, sometimes the first letter is capitalized and sometimes not; please harmonize. It would be more appropriate if the retention test followed the post-test in a linear way (by enlarging the figure and continuing on the next page). The phrase “After 6 classes” is misplaced, as it gives the impression that six classes occur between the end of the protocol and the posttest session.**

Your suggestion is very good. We have made modifications to the figure.

**Learning outcome data collection**

**Line 389 and….: Could you provide examples of items or questions for each of the scales (motor cognition, movement result performance...)?**

Thank you for your revision suggestions. Due to limited space, we have chosen to provide examples to illustrate. In this experiment, the learning information provided in the video materials was consistent with both the Set Shot Movement Pattern Evaluation Scale and the Set Shot Motor Cognition Test, establishing alignment across learning materials, motor skill assessments, and motor cognition evaluations.

For example, the key point: In the preparation stage, the ball should be held between the chin and the chest. This is included as part of the multimedia teaching materials during instruction. It also appears as a multiple-choice question in the Set Shot Motor Cognition Test (e.g., "During the preparation stage, where should the ball be held?"). In the Movement Pattern Performance assessment, this is a scoring criterion. Expert raters observe the students' movements and evaluate them based on the key technical points. （Lines：393-398）

**Results :**

**It would seem more appropriate to present the results in the form of graphs rather than tables.**

We thank the reviewer for the suggestion. We understand the advantages of figures in terms of visualization. Considering that the results of this study involve numerical comparisons across multiple dimensions (such as movement staging and movement limb segments), we believe that tables are currently the form that can most clearly and accurately present this structured data. We have already provided focused interpretation of the key findings from the tables in the main text to aid reader comprehension.

**Discussion :**

**Line 665-672 : Be careful in your interpretation, this remains a hypothesis.**

**Line 673-683 : The authors argue that visual cues enhance learning by guiding attention. Given that no direct measures of attention (e.g., eye-tracking, subjective scales) were used, this limits the robustness of the interpretations. I suggest qualifying the conclusions on this point and stating more explicitly that these results remain hypothetical.**

The above suggestions have been revised. Thank you again for your feedback.

This finding lends support to the hypothesis that…… （Lines：676）

this study hypothesized that……（Lines：685）

**Line 676 : « For example, Zhang et al. » the date is missing after the reference**

The correction has been completed.

**Line 649 : You discuss multimodality as the contribution of complementary information, but in itself multimodality can improve motor performance and learning, as seen in these papers :**

Thank you very much to the reviewer for providing many references. After reading, we have selected the following references for use.

Research has demonstrated that consistent audiovisual stimuli can facilitate procedural perceptual-motor learning[51]. （Lines：627-629）

Research showed that multisensory stimulation not only facilitates early perceptual processing and motor responses but also enhances higher cognitive processes such as information discrimination and response selection [63].（Lines：757-759）

51. Lagarrigue Y, Cappe C, Tallet J. Regular rhythmic and audio-visual stimulations enhance procedural learning of a perceptual-motor sequence in healthy adults: A pilot study. PLoS One. 2021;16(11): e0259081.

63. Hecht D, Reiner M, Karni A. Multisensory enhancement: gains in choice and in simple response times. Exp Brain Res. 2008 May 14;189(2):133.
